# Perioperative Predictors of Early Spinal Cord Stimulator Removal: A Retrospective Cohort Study

**DOI:** 10.3390/neurolint17070100

**Published:** 2025-06-27

**Authors:** Peyton J. Murin, Patrick J. Murin, Sejal V. Jain, Yuri Chaves Martins

**Affiliations:** 1 Department of Neurology, Saint Louis University School of Medicine, St. Louis, MO 63104, USA; peyton.murin@slucare.ssmhealth.com; 2 Department of Psychology, Rhodes College, Memphis, TN 38112, USA; murpj-27@rhodes.edu; 3 Department of Anesthesiology and Pain Management, The University of Texas, Southwestern Medical Center, Dallas, TX 75390, USA; sejal.jain@utsouthwestern.edu; 4 Department of Anesthesiology, Saint Louis University School of Medicine, St. Louis, MO 63110, USA

**Keywords:** spinal cord stimulator, chronic pain management, explantation, surgical indication, degenerative spine disease

## Abstract

Background: Spinal cord stimulators can offer an effective treatment in chronic pain refractory to conventional medical management. However, with a failure rate of up to 44% and an annual explantation rate of 6–9%, there is a need to better identify patients at high risk for therapeutic failure. The objective of this retrospective cohort study was to determine predictors of early SCS explantation following device placement. Methods: The Medical Informatics Operating room Vitals and Events Repository database was queried for patients with a spinal cord stimulator and at least two years of follow-up (*n* = 56). A multivariate logistic regression was fitted. Recursive factor elimination with cross-validation and L1 penalization were used to reduce the number of predictors and minimize the risk of overfitting. The model was used to predict risk factors for explantation, odds ratio (OR), 95% confidence interval (CI), and false discovery rate-adjusted *p*-value. Results: The final model displayed adequate performance with an average precision of 0.769. Sleep disorders were identified as a statistically significant predictor of SCS explantation (OR: 3.88, CI: 1.36–11.04, FDR *p*-value: 0.0497). Conclusions: While further prospective studies are needed, our study indicates that sleep disorders are a risk factor for spinal cord stimulator explantation and should be considered during pre-operative evaluation.

## 1. Introduction

Chronic pain represents a significant public health challenge, and, for some patients who fail conventional treatment options, spinal cord stimulator (SCS) trial and implantation offers an effective neuromodulation strategy [1]. Despite its efficacy, SCS explantation remains a persistent concern, with previous studies estimating explantation rates of 6–38% [2,3,4,5]. This not only impacts patient satisfaction but also contributes to an increased financial burden on healthcare systems.

Evidence on the incidence of and risk factors associated with SCS explantation is limited by heterogeneous patient populations and mixed findings [4,6,7]. Moreover, while therapeutic failure, commonly defined as an inability to achieve at least 50% pain relief, has been reported in up to 44% of SCS recipients, the factors driving device removal remain poorly understood [8,9]. Notably, the rate of explantation appears to increase with time since implantation, though the trajectory of this trend and the associated patient characteristics are not well defined [5,8].

This retrospective cohort study seeks to analyze data from a large-scale electronic health record database to identify factors associated with early SCS explantation—defined as explantation for any reason besides end of battery life within two years of device placement. We used a multivariate logistic regression model to exam how patient-specific factors influence the risk of early SCS explantation.

## 2. Materials and Methods

The study was designed in accordance with TRIPOD-AI guidelines for reporting clinical prediction models that use regression or machine learning methods.

### 2.1. Data

Data were collected from the Medical Informatics Operating room Vitals and Events Repository (MOVER) database containing electronic health records (EHRs) from 58,799 unique patients who underwent surgery at the University of California Irvine Medical Center (UCI) between January 2018 and July 2023 [10]. The data were compiled by the UCI investigators using an original retrospective review of the EHR and, where applicable, waveform matching. Patient health information (PHI) was manually removed from free text, patient age capped at 90, and dates shifted by a consistent, random number of days to comply with the HIPAA Privacy Rule. A data use agreement (DUA) was completed to allow access to the data. The database is organized by patient ID, with comprehensive EHR data comprising patient demographics, medical comorbidities, surgical procedure, operative medicines, lines, drains, and any post-operative complications included for each patient. The UCI MOVER study team conducts maintenance on and updates of the database.

### 2.2. Participants

**Figure 1:**
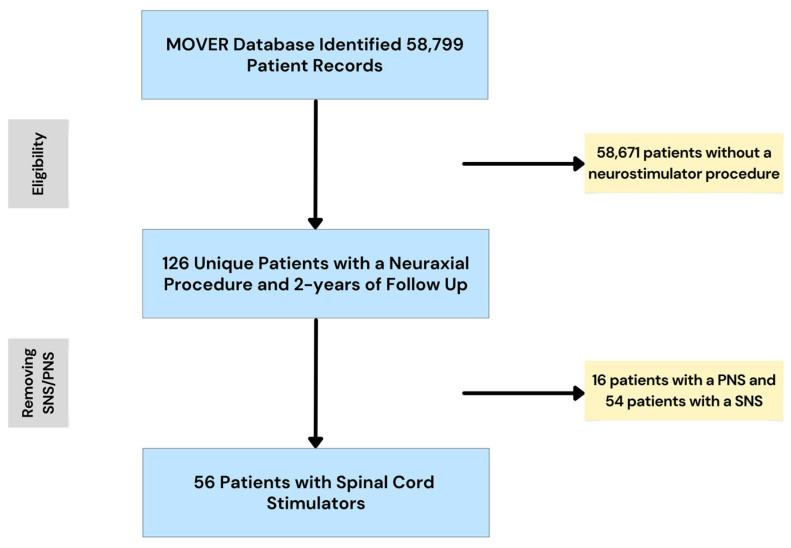
The MOVER database contained 58,799 patient records, including 126 unique patients with a neuraxial procedure and at least 2 years of follow-up. Of these 126 patients, 56 underwent SCS implantation. SNS = sacral nerve stimulator. PNS = peripheral nerve stimulator.

Patients included in the study were patients undergoing SCS implantation at a single center (UCI). The treatment received was an implantation of an SCS. Inclusion criteria: adult (≥18 years of age), SCS procedure, and ≥2 years of follow-up. Exclusion criteria: <18 years of age, <2 years of follow-up, peripheral nerve stimulator (*n* = 16), and sacral nerve stimulator (*n* = 54). Following the review of the database and application of the inclusion/exclusion criteria, we were left with a study cohort of 56 unique patients with SCS implantation and at least 2 years of follow-up. Figure 1 illustrates the sampling process.

### 2.3. Data Preparation

Study variables were created using the International Classification of Diseases (ICD)-9-CM codes, procedure codes/claims (ICD-9, -10, and CPT), demographic data, the American Society of Anesthesiologist (ASA) score, anesthesia type, and post-operative events, including hospitalizations and intensive care unit (ICU) admissions. The included codes and/or definitions for each of the study variables can be seen in Table S1. Sex was encoded as 1: female; 0: male. Medical comorbidities were encoded as 1: present; 0: absent. Anesthesia type was encoded as 1: monitored anesthesia care; 0: general anesthesia. ICU admission was encoded as 1: yes; 0: no. The LOS and ASA score was encoded as the numerical value. Explantation was defined as SCS removal within the first two years following placement for any reasons other than end of battery life. A good outcome was defined as no SCS removal within the first two years following implantation.

### 2.4. Predictors

Predictors were chosen broadly based upon the prior literature reports [7,11,12,13] and author clinical experience. We chose to err on the side of a broad inclusion of variables, as our methodology used recursive feature elimination with cross-validation (RFECV), employed an L1-penalized logistic regression, and allowed for removal of unstable predictor variables.

### 2.5. Sample Size

All patients meeting inclusion/exclusion criteria were included. The study cohort consisted of 56 patient records.

### 2.6. Missing Data

Two records were excluded for missing data. A formal sensitivity analysis was not conducted; however, given the extremely small proportion (<4%) of excluded data, the potential for exclusion bias is likely minimal.

### 2.7. Analytical Methods

Age and length of stay were reported as means ± standard deviation (SD) and the ASA score was reported as median with interquartile range (IQR). All categorical variables were reported as percentages. First, Fisher’s exact test (categorical variables) and the Mann–Whitney U test (numerical variables) were used to compare patients with early explantation to those without early explantation. Statistical significance was set at *p* < 0.05. Given the limited associations previously identified using basic statistical methods, we sought to use a logistic regression model which could account for interactions between variables. Data were imported into Python 3.10. (Anaconda Software Distribution, Austin, TX, USA) with the following add-ons used for analysis: pandas24 [14], numpy [15], scikit-learn [16], sklearn.linear_model [17], imblearn [16], matplotlib [14], and seaborn [18]. The outcome variable was defined as explantation = 1 or no explantation = 0. Values were standardized via z-score normalization (StandardScaler). The dataset was divided into training and test sets using stratified random sampling (80/20 split). Given the low number of outcomes (*n* = 14) and the high number of predictor variables, we sought to reduce the number of predictors to identify an optimal feature set using RFECV, employing an L1-penalized logistic regression (LogisticRegression with penalty = ‘L1’, solver = ‘saga’) as the base estimator and 5-fold cross-validation. The optimal feature subset was identified based on the area under the curve—receiver operating characteristic (AUC–ROC).

A multivariable logistic regression model was then fit using statsmodels. In cases where matrix singularity was encountered, L1-regularized regression (fit_regularized) was used as a fallback. Odds ratios (ORs), 95% confidence intervals (CIs), and false discovery rate-adjusted (FDR, Benjamini–Hochberg method) *p*-values were reported. The eight most frequently selected features were identified across iterations using a feature importance counter. The final model was evaluated on the original dataset using the average precision score (AP).

### 2.8. Class Imbalance

To address class imbalance, synthetic minority oversampling technology (SMOTE) was applied to the training set only, preventing information leakage.

### 2.9. Fairness

To ensure model fairness, minimize the risk of overfitting, and accurately assess model performance, we applied recursive factor elimination with cross-validation combined with an L1-penalized logistic regression and reported FDR-adjusted *p*-values.

### 2.10. Model Output

Model output consisted of odds ratios, 95% confidence intervals, and false discovery rate-adjusted *p*-values. In rare outcomes, such as SCS explantation, the odds ratio approximates relative risk. Odds ratios and 95% confidence intervals >1.0 or <1.0 were considered statistically significant.

## 3. Results

### 3.1. Cohort Demographics

Baseline cohort characteristics are shown in Table 1. The overall cohort was 55.4% female with an average age of 60.0 (+/−14.2) years. The most common indications for SCS implantation were low back pain (69.6%) and failed back surgery syndrome (16.1%). The median ASA score was 3.0 (IQR: 2–3), and the cases were predominantly done under general anesthesia (78.6%) as opposed to monitored anesthesia care (MAC). The average length of stay following implantation was 0.7 (+/−0.3) days, with no statistically significant difference seen in patients with explantation compared with those without explantation. The most common medical comorbidities were hypertension (26.8%) and hyperlipidemia (25.0%), followed by sleep disorder (17.9%), arthritis (17.9%), musculoskeletal pain (17.9%), and depression (17.9%). The reasons for explantation were SCS generator of lead dysfunction (*n* = 6), infection (*n* = 3), and CSF leak (*n* = 1). For four patients, no reason was documented. There were no statistically significant differences in demographic or medical comorbidities between the cohorts by Fisher’s exact test analysis.

### 3.2. Multivariable Logistic Regression Model

Given the limited associations identified by basic statistical analysis, we hypothesized that an effective predictive model would need to be able to account for the interactions between multiple variables. To this end, we utilized a multivariable logistic regression model. Combining recursive factor elimination with cross-validation, eight candidate predictor factors were identified: cardiovascular disease, sleep disorders, hypertension, atrial fibrillation, diabetes mellitus, urinary dysfunction, ICU admission, and female sex. The odds ratios, confidence intervals, and FDR-adjusted *p*-values can be seen in Table 2.

**Figure 2:**
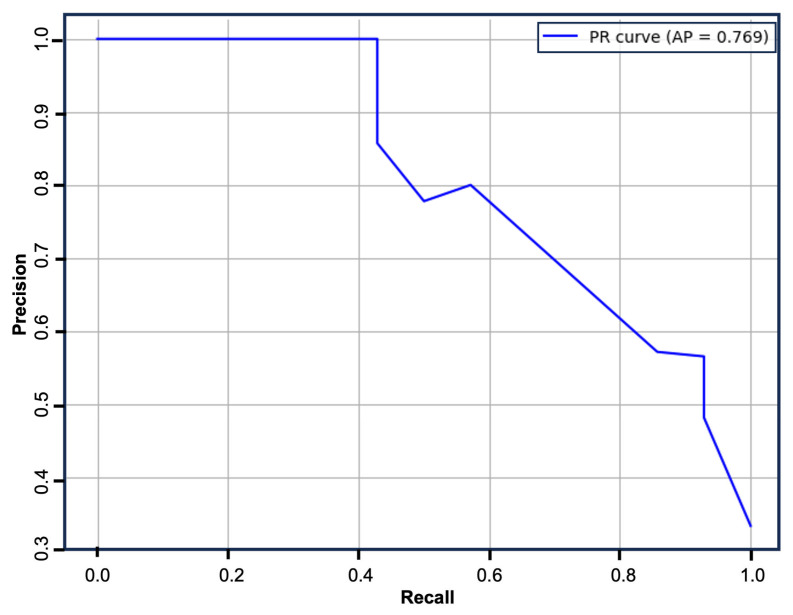
Precision–recall curve. The multivariable logistic regression model displayed good performance with an AP of 0.769.

The logistic regression model was refitted using the above predictors and assessed on the complete dataset. The model displayed good performance with an AP of 0.769 (Figure 2).

**Figure 3:**
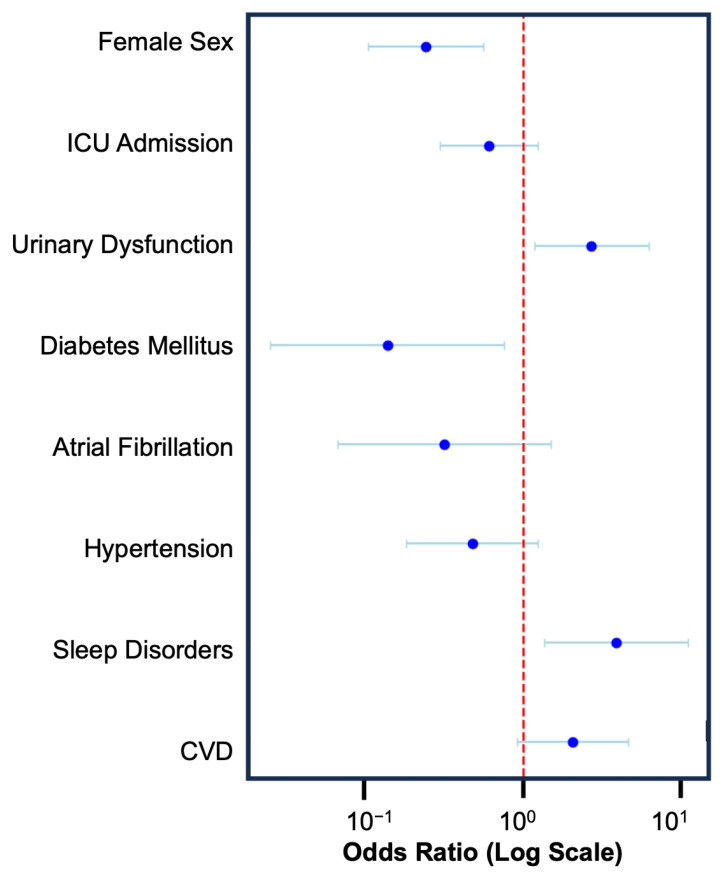
Forest plot illustrating the odds ratio and 95% confidence interval for the eight factors included in the multivariable logistic regression model. Sleep disorders (OR: 3.88, CI: 1.36–11.04) conveyed an increased risk of explantation, and female sex (OR: 0.24, CI: 0.10–0.56) conveyed a decreased risk of explantation.

Amongst the assessed variables, three were predictive of increased risk of explantation: cardiovascular disease (OR: 2.04, CI: 0.90–4.59), sleep disorders (OR: 3.88, CI: 1.36–11.04), and urinary dysfunction (OR: 2.69, CI: 1.17–6.19). Hypertension, atrial fibrillation, diabetes mellitus, ICU admission, and female sex were included in the model; however, all conferred a decreased risk of explantation (Figure 3). Only sleep disorders (FDR *p*-value: 0.4969) and female sex (FDR *p*-value: 0.0081) met the threshold for statistical significance after correction for the false discovery rate.

## 4. Discussion

SCS can offer an effective therapeutic option in patients with chronic pain refractory to conventional treatment [1]. However, early explantation remains a significant problem, with explantation rates of 6–9% [2,4,5]. In this single-center retrospective cohort study, we fitted a logistic regression model capable of predicting explantation with adequate performance. In doing so, we identified sleep disorders as a potential risk factor for SCS explantation. Our findings suggest sleep disorders may predispose patients to suboptimal outcomes following SCS implantation and should therefore be considered in the pre-operative evaluation.

We hypothesize that the observed association between sleep disorders and SCS explantation may be attributable to an increased predisposition to central sensitization, heightened systemic inflammation, and diminished pain thresholds in this population of patients, all of which contribute to poor pain modulation [19,20]. In patients with sleep-disordered breathing, there is a state of chronic hypoxia resulting in oxidative stress and increased levels of inflammatory mediators [20]. Increase levels of inflammatory mediators such as Interleukin-6 and Tumor Necrosis Factor-alpha have also been shown in patients with sleep disorders [21]. As such, we hypothesize that sleep disorders may cause increased nociceptive sensitivity and decreased pain tolerance, resulting in poor response to SCS therapy. In addition, we propose that adequate management of any existing sleep disorders prior to and after SCS implantation could improve SCS outcomes.

Interestingly, female sex was protective against explantation. While basic statistics noted no differences between the cohorts in gender distribution between patients with explantation or no explantation, the multivariable logistic regression noted female sex to convey a decreased risk. The association between SCS outcomes and biological sex is not fully understood [22,23,24,25]. Female sex has recently been associated with a small increased risk of SCS explantation [23]. However, previous studies showed comparable response and outcomes to SCS therapy in males and females, despite higher baseline pain scores in the female patients [22,24,25]. As there are differences in pain biology between males and females [26], it is possible that these differences may result in different responses to SCS therapy. Therefore, further studies are needed to assess the validity of this finding and to determine a possible pathophysiological explanation.

Previous studies attempted to identify factors associated with SCS explantation. A retrospective study using univariate logistic regression identified comorbid depression, pre- or post-operative opioid use, cannabis use, tobacco use, and comorbid coagulopathy as risk factors for explantation [4]. Another study did a similar univariate regression analysis, noting an association between any psychiatric comorbidities and an increased risk of any complications, infections, lead displacements, surgical pain, explantations, and one-year readmission rates [27]. Unlike the previous study, the authors followed up this analysis with a multivariate logistic regression, noting an increased risk of any complications, reoperations, or readmissions with each additional psychiatric comorbidity. A smaller study of 253 patients in a private health insurance database applied a bivariate analysis approach, identifying younger age, tobacco use, and the presence of other mental health disorders—defined as any mental health diagnosis except depression or anxiety—as risk factors for explantation [7]. A fourth study, using local and national registries in Sweden, identified the use of 10 kHz versus tonic waveform and age 60 years or older as risk factors, while higher education and being employed were associated with a good outcome [9].

While our results are promising, with an AP of 0.769, the associations should be interpreted with caution. The use of a single-center design and a claims-based dataset limited the ability to capture patient-reported outcomes, granular clinical reasoning, and device-specific factors such as SCS programming [28]. Furthermore, many steps were taken to minimize the risk of overfitting; however, the small sample size (*n* = 56) and retrospective design limited the generalizability. While our model demonstrates reasonable internal validity, its generalizability to other institutions and healthcare systems remains untested. Future efforts will focus on external validation using multicenter datasets or national registries to ensure performance consistency across diverse patient populations and care settings. We also chose a two-year follow-up as the cutoff; however, it is important to note that prior work has noted no time effect on the incidence of SCS explantation, infection, or lead/generator dysfunction [29]. It is expected that a certain portion of patients were lost to follow-up, with the prior literature showing that approximately 19% of SCS patients are typically lost to follow-up. However, the most common reason for loss to follow-up in previous studies was improvement in pain (58% of patients), suggesting these patients are most likely to represent a favorable rather than adverse outcome [30].

## 5. Conclusions

SCS remains a valuable therapeutic option compared with conventional medical management [31]. With ongoing efforts to expand SCS indications and utilization [27], it remains important to identify those patients most likely to benefit and those at high risk for explantation. In this single-center retrospective cohort study, we pilot a novel analysis technique, identifying sleep disorders as a potential risk factor for SCS explantation. Further prospective studies with larger sample sizes are needed to validate these findings.

## Figures and Tables

**Table 1:** Comparison of demographic, perioperative, and medical comorbidity variables.

Variable	Overall	Explantation	No Explantation	*p*-Value
Number of Patients	56	14	42	
Sex				
Male *n* (%)	25 (44.6%)	7 (50.0%)	18 (42.9%)	0.7593
Female *n* (%)	31 (55.4%)	7 (50.0%)	24 (57.1%)	0.7593
Age (years ± SD)	60.0 ± 14.2	62.6 ± 13.7	59.1 ± 14.5	0.2735
ASA Score (IQR)	3.0 (2–3)	3.0 (2–3)	3.0 (2–3)	0.3613
Anesthesia Type				
Monitored Anesthesia Care *n* (%)	12 (21.4%)	3 (21.4%)	9 (21.4%)	>0.9999
General Anesthesia *n* (%)	44 (78.6%)	11 (78.6%)	38 (78.6%)	>0.9999
SCS Lead Type				
Paddle Lead *n* (%)	31 (55.4%)	4 (28.6%)	19 (45.2%)	>0.9999
Percutaneous Lead *n* (%)	13 (23.2%)	2 (14.3%)	8 (19.0%)	>0.9999
Unspecified *n* (%)	12 (21.4%)	8 (57.1%)	15 (35.7%)	N/A
Length of Stay (days ± SD)	0.7 ± 0.3	2.4 ± 5.5	0.6 ± 0.9	0.1632
ICU Admission *n* (%)	4 (7.1%)	1 (7.1%)	3 (7.1%)	>0.9999
Possible Indications for SCS *				
Failed Back Surgery *n* (%)	9 (16.1%)	3 (21.4%)	6 (14.3%)	0.6759
Peripheral Neuropathy *n* (%)	7 (12.5%)	1 (7.1%)	6 (14.3%)	0.6662
Low Back Pain *n* (%)	39 (69.6%)	8 (57.1%)	31 (73.8%)	0.3171
Cervical Pain *n* (%)	8 (14.3%)	2 (14.3%)	6 (14.3%)	>0.9999
Urinary Dysfunction *n* (%)	8 (14.3%)	3 (21.4%)	5 (11.9%)	0.3981
Past Medical History				
Cerebrovascular Disease	2 (3.6%)	1 (7.1%)	1 (2.4%)	0.4409
Obstructive Sleep Apnea *n* (%)	8 (14.3%)	3 (21.4%)	5 (11.9%)	0.3981
Sleep Disorder *n* (%)	10 (17.9%)	4 (28.6%)	6 (14.3%)	0.2472
Hypertension *n* (%)	15 (26.8%)	3 (21.4%)	12 (28.6%)	0.7364
Hyperlipidemia *n* (%)	14 (25.0%)	5 (35.7%)	9 (21.4%)	0.3045
Atrial Fibrillation *n* (%)	5 (8.9%)	0 (0.0%)	5 (11.9%)	0.3163
Diabetes Mellitus *n* (%)	6 (10.7%)	0 (0.0%)	6 (14.3%)	0.3195
Chronic Kidney Disease *n* (%)	3 (5.4%)	2 (14.3%)	1 (2.4%)	0.1510
Anxiety *n* (%)	7 (12.5%)	1 (7.1%)	6 (14.3%)	0.6662
Depression *n* (%)	10 (17.9%)	1 (7.1%)	9 (21.4%)	0.4226
Fibromyalgia *n* (%)	3 (5.4%)	2 (14.3%)	1 (2.4%)	0.1510
Irritable Bowel Syndrome *n* (%)	3 (5.4%)	2 (14.3%)	1 (2.4%)	0.1510
Obesity *n* (%)	8 (14.3%)	4 (28.6%)	4 (9.5%)	0.0970
Migraine *n* (%)	2 (3.6%)	1 (7.1%)	1 (2.4%)	0.4409
Musculoskeletal Pain *n* (%)	10 (17.9%)	3 (21.4%)	7 (16.7%)	0.6984
Arthritis *n* (%)	10 (17.9%)	5 (35.7%)	5 (11.9%)	0.0998
Malignancy *n* (%)	5 (8.9%)	2 (14.3%)	3 (7.1%)	0.5898
Social History				
Opioid Use *n* (%)	7 (12.5%)	2 (14.3%)	5 (11.9%)	>0.9999
Illicit Substance Use *n* (%)	1 (1.8%)	0 (0.0%)	1 (2.4%)	>0.9999
Tobacco Products *n* (%)	3 (5.4%)	0 (0.0%)	3 (7.1%)	0.5652

The parametric *p*-value is calculated by the Fisher’s exact test for categorical variables. The non-parametric *p*-value is calculated by the Mann–Whitney U test for numerical values. * In patients with multiple listed indications, they were included for each of the indications.

**Table 2:** Potential predictor variables.

Variable	Odds Ratio	Lower CI	Upper CI	*p*-Value	FDR-Adjusted *p*-Value
Cardiovascular Disease	2.0385	0.9047	4.5932	0.0857	0.1286
Sleep Disorders	3.8792	1.3636	11.0355	0.0110	0.0497
Hypertension	0.4709	0.1795	1.2357	0.1260	0.1620
Atrial Fibrillation	0.3138	0.0659	1.4939	0.1455	0.1636
Diabetes Mellitus	0.1368	0.0248	0.7559	0.0226	0.0507
Urinary Dysfunction	2.6935	1.1718	6.1916	0.01963	0.0507
ICU Admission	0.6039	0.2953	1.2349	0.1670	0.1670
Female Sex	0.2388	0.1025	0.5562	0.0009	0.0081

CI = confidence interval. FDR = false discovery rate.

## Data Availability

The data that supports the findings of this study are openly available in the MOVER database at https://mover.ics.uci.edu/index.html (accessed on 26 June 2025), reference [10]. Analytical code is available on request.

## Supplementary Materials

The following supporting information can be downloaded at: https://www.mdpi.com/article/10.3390/neurolint17070100/s1, Table S1: Data collection sheet describing how each variable was defined, relevant ICD-9 codes, and relevant CPT codes.

## Abbreviations

The following abbreviations are used in this manuscript:

ASA	American Society of Anesthesiologists
AUC–ROC	Area Under the Curve—Receiver Operating Characteristic
CI	Confidence Interval
CPT	Current Procedural Terminology
EHR	Electronic Health Record
FDR	False Discovery Rate
HIPAA	Health Insurance Portability and Accountability Act
ICD	International Classification of Diseases
ICU	Intensive Care Unit
IQR	Interquartile Range
LOS	Length of Stay
MAC	Monitored Anesthesia Care
MOVER	Medical Informatics Operating room Vitals and Events Repository
OR	Odds Ratio
PHI	Protected Health Information
PNS	Peripheral Nerve Stimulator
RFECV	Recursive Feature Elimination with Cross-Validation
SCS	Spinal Cord Stimulator/Spinal Cord Stimulation
SD	Standard Deviation
SMOTE	Synthetic Minority Oversampling Technique
SNS	Sacral Nerve Stimulator

## References

[1] Thomson S., Huygen F., Prangnell S., De Andres J., Baranidharan G., Belaid H., Berry N., Billet B., Cooil J., De Carolis G. (2020). Appropriate referral and selection of patients with chronic pain for spinal cord stimulation: European consensus recommendations and e-health tool. Eur. J. Pain.

[2] Blackburn A.Z., Chang H.H., DiSilvestro K., Veeramani A., McDonald C., Zhang A.S., Daniels A. (2021). Spinal Cord Stimulation via Percutaneous and Open Implantation: Systematic Review and Meta-Analysis Examining Complication Rates. World Neurosurg..

[3] Gatzinsky K., Brink B., Eygloardottir K.L., Hallen T. (2024). Long-term explantation risk in patients with chronic pain treated with spinal cord or dorsal root ganglion stimulation. Reg. Anesth. Pain Med..

[4] Hussain N., Boulos R., Malik T.M., Abd-Elsayed A., Essandoh M.K., Khan S., Nguyen A., Weaver T.E. (2023). Identifying Predictors for Early Percutaneous Spinal Cord Stimulator Explant at One and Two Years: A Retrospective Database Analysis. Neuromodulation.

[5] Rauck R.L., Loudermilk E., Thomson S.J., Paz-Solis J.F., Bojrab L., Noles J., Vesper J., Atallah J., Roth D., Hegarty J. (2023). Long-term safety of spinal cord stimulation systems in a prospective, global registry of patients with chronic pain. Pain Manag..

[6] Bir S.C., Konar S., Maiti T., Nanda A., Guthikonda B. (2016). Neuromodulation in intractable pain management: Outcomes and predictors of revisions of spinal cord stimulators. Neurosurg. Focus.

[7] Dougherty M.C., Woodroffe R.W., Wilson S., Gillies G.T., Howard M.A., Carnahan R.M. (2021). Risk Factors and Survival Analysis of Spinal Cord Stimulator Explantation. Neuromodulation.

[8] Al-Kaisy A., Royds J., Al-Kaisy O., Palmisani S., Pang D., Smith T., Padfield N., Harris S., Wesley S., Yearwood T.L. (2020). Explant rates of electrical neuromodulation devices in 1177 patients in a single center over an 11-year period. Reg. Anesth. Pain Med..

[9] Kirketeig T., Soreskog E., Jacobson T., Karlsten R., Zethraeus N., Borgstrom F. (2023). Real-world outcomes in spinal cord stimulation: Predictors of reported effect and explantation using a comprehensive registry-based approach. Pain Rep..

[10] Samad M., Angel M., Rinehart J., Kanomata Y., Baldi P., Cannesson M. (2023). Medical Informatics Operating Room Vitals and Events Repository (MOVER): A public-access operating room database. JAMIA Open.

[11] Mekhail N., Azer G., Saweris Y., Mehanny D.S., Costandi S., Mao G. (2018). The Impact of Tobacco Cigarette Smoking on Spinal Cord Stimulation Effectiveness in Chronic Spine-Related Pain Patients. Reg. Anesth. Pain Med..

[12] Patel S.K., Gozal Y.M., Saleh M.S., Gibson J.L., Karsy M., Mandybur G.T. (2020). Spinal cord stimulation failure: Evaluation of factors underlying hardware explantation. J. Neurosurg. Spine.

[13] Murin P.J., Murin P.J., Lima de Mendonca Y., Martins Y.C. (2025). Identification of Perioperative Risk Factors for Early Sacral Nerve Stimulator Explantation: A Single-Center Retrospective Cohort Study. J. Clin. Med..

[14] Hunter J.D. (2007). Matplotlib: A 2D graphics environment. Comput. Sci. Eng..

[15] Harris C.R., Millman K.J., van der Walt S.J., Gommers R., Virtanen P., Cournapeau D., Wieser E., Taylor J., Berg S., Smith N.J. (2020). Array programming with NumPy. Nature.

[16] Guillaume Lemaıtre F.N., Christos K. (2017). Aridas. Imbalanced-learn: A Python Toolbox to Tackle the Curse of Imbalanced Datasets in Machine Learning. J. Mach. Learn. Res..

[17] Pedregosa F., Varoquaux G., Gramfort A., Michel V., Thirion B., Grisel O., Blondel M., Prettenhofer P., Weiss R., Dubourg V. (2011). Scikit-learn: Machine Learning in Python. J. Mach. Learn. Res..

[18] Virtanen P., Gommers R., Oliphant T.E., Haberland M., Reddy T., Cournapeau D., Burovski E., Peterson P., Weckesser W., Bright J. (2020). SciPy 1.0: Fundamental algorithms for scientific computing in Python. Nat. Methods.

[19] Jain S.V., Panjeton G.D., Martins Y.C. (2024). Relationship Between Sleep Disturbances and Chronic Pain: A Narrative Review. Clin Pract..

[20] Besedovsky L., Lange T., Haack M. (2019). The Sleep-Immune Crosstalk in Health and Disease. Physiol. Rev..

[21] Ng S.-M., Yin M.X., Chan J.S., Chan C.H., Fong T.C., Li A., So K.-F., Yuen L.-P., Chen J.-P., Chung K.-F. (2022). Impact of mind–body intervention on proinflammatory cytokines interleukin 6 and 1β: A three-arm randomized controlled trial for persons with sleep disturbance and depression. Brain Behav. Immun..

[22] Bretherton B., de Ridder D., Crowther T., Black S., Whelan A., Baranidharan G. (2022). Men and Women Respond Equally Well to Spinal Cord and Dorsal Root Ganglion Stimulation. Neuromodulation.

[23] Grabnar M., Wilson R. (2025). Sex Differences in Rates of Spinal Cord Stimulation Therapy and Spinal Cord Stimulator Explants: A Propensity-Score Matched Analysis. Neuromodulation.

[24] Conic R.R., Caylor J., Cui C.L., Reyes Z., Nelson E., Yin S., Lerman I. (2022). Sex-specific differences in the efficacy of traditional low frequency versus high frequency spinal cord stimulation for chronic pain. Bioelectron. Med..

[25] Mekhail N., Costandi S., Saweris Y., Armanyous S., Chauhan G. (2022). Impact of biological sex on the outcomes of spinal cord stimulation in patients with chronic pain. Pain Pract..

[26] Mogil J.S., Parisien M., Esfahani S.J., Diatchenko L. (2024). Sex differences in mechanisms of pain hypersensitivity. Neurosci. Biobehav. Rev..

[27] Beletsky A., Liu C., Alexander E., Hassanin S.W., Vickery K., Loomba M., Winston N., Chen J., Gabriel R.A. (2023). The Association of Psychiatric Comorbidities With Short-Term and Long-Term Outcomes Following Spinal Cord Stimulator Placement. Neuromodulation.

[28] Hussain N., Weaver T. (2023). Response to the Letter to the Editor Regarding: “Identifying Predictors for Early Percutaneous Spinal Cord Stimulator Explant at One and Two Years: A Retrospective Database Analysis”. Neuromodulation.

[29] Goudman L., Moens M., Kelly S., Young C., Pilitsis J.G. (2024). Incidence of Infections, Explantations, and Displacements/Mechanical Complications of Spinal Cord Stimulation During the Past Eight Years. Neuromodulation.

[30] Kang K., Glicksman M., Ho J., Hoang K., Phung A., Madabhushi S., Hasoon J., Yazdi C., Fonseca A.C., Kaye A.D. (2024). Single Institutional Cross-Sectional Phone Survey Study: Evaluation of Causes for Loss to Follow-up After Spinal Cord Stimulator Implantation. Pain Physician.

[31] Huygen F., Soulanis K., Rtveladze K., Kamra S., Schlueter M. (2024). Spinal Cord Stimulation vs. Medical Management for Chronic Back and Leg Pain: A Systematic Review and Network Meta-Analysis. JAMA Netw. Open.

