# Supplementary material for: Perioperative Predictors of Early Spinal Cord Stimulator Removal: A Retrospective Cohort Study
Source: Neurol Int. 2025 Jun 27;17(7):100. doi: 10.3390/neurolint17070100 (PMC12299350; doi:10.3390/neurolint17070100)
Supplement: Supplementary file 1 [file neurolint-17-00100-s001.zip › neurolint-3681704-supplementary.pdf]

Table S1: Data collection sheet describing how each variable was defined, relevant ICD-9 codes, and relevant CPT codes.

**Variable**	**Definition**
Age at time of implant	Age in years
Gender	Female: 1, Male: 0
Paddle SCS implant surgery	CPT: 63655<br>ICD: 00HU0MZ, 00HV0MZ
Percutaneous SCS implant surgery	CPT: 63650
Unspecified SCS implant surgery	Procedure name only
SCS explantation	CPT: 63662, 63688
ASA score	American Society of Anesthesiologist Score (1-5)
Length of Stay	Length of stay in days following implantation for any reason.
ICU Admission	Admission to the ICU following implantation for any reason.
Past Medical History	Medical comorbidity data will be queried using ICD-9 codes, with assessed diagnoses including cerebrovascular disease (ICD 429.2), failed back surgery syndrome (ICD 722.83), peripheral neuropathy (ICD 724.2), neck pain (ICD 723.1), obstructive sleep apnea (OSA) (ICD 327.23), sleep disorders (ICD: 780.52, 327.23, 307.42, 327.01), hypertension (ICD 401.9), hyperlipidemia (ICD: 272.4), atrial fibrillation (ICD: 427.31), type 2 diabetes mellitus (ICD 250), chronic kidney disease (CKD) (ICD: 585.9), anxiety (ICD: 300), depression (ICD: 296.2), attention deficit-hyperactivity disorder (ADHD) (ICD: 314.01), fibromyalgia (ICD 729.1), irritable bowel syndrome (IBS) (ICD: 564.1), obesity (ICD: 278, OR Last BMI on/before SCS implant is ≥ 30), migraine (ICD: 346), urinary dysfunction (ICD 788.69), opioid use (ICD: 304, 292.9, V58.83), substance use (ICD: 305.9), alcohol abuse (ICD: 305, 790.3), tobacco use (ICD: V15.82, 305.1), muscle pain (ICD: 729.1), arthritis (ICD: 711, 715, 716), obsessive compulsive disorder (OCD) (ICD: 300.3), post-traumatic stress disorder (PTSD) (ICD: 309.81) and malignancy (ICD: 199.1, 275.42).

SCS: Spinal cord stimulator
